# A horse or a zebra? Unusual manifestations of common cutaneous infections in primary immunodeficiency pediatric patients

**DOI:** 10.3389/fped.2023.1103726

**Published:** 2023-03-06

**Authors:** Ayelet Ollech, Amos J Simon, Atar Lev, Tali Stauber, Gilad Sherman, Michal Solomon, Aviv Barzilai, Raz Somech, Shoshana Greenberger

**Affiliations:** ^1^ Department of Dermatology, Pediatric Dermatology Service, Sheba Medical Center, Tel-Hashomer, Ramat-Gan, Israel; ^2^ Sackler Faculty of Medicine, Tel Aviv University, Tel Aviv, Israel; ^3^ Sheba Cancer Research Center and Institute of Hematology, Sheba Medical Center, Tel-Hashomer, Ramat-Gan, Israel; ^4^ Pediatric Department A and the Immunology Service, Jeffrey Modell Foundation Center, Edmond and Lily Safra Children's Hospital, Sheba Medical Center, Tel-Hashomer, Ramat-Gan, Israel; ^5^ Pediatric Infectious Disease Unit, Edmond and Lily Safra Children's Hospital, Sheba Medical Center, Tel-Hashomer, Ramat-Gan, Israel; ^6^ Department of Dermatology, Sheba Medical Center, Tel-Hashomer, Ramat-Gan, Israel

**Keywords:** immunological aspects, cutaneous, infection, inborn error of immunity, pediatric, primary immunodeficiencies

## Abstract

**Background:**

Patients with primary immunodeficiency disorders (PIDs) often suffer from recurrent infections because of their inappropriate immune response to both common and less common pathogens. These patients may present with unique and severe cutaneous infectious manifestations that are not common in healthy individuals and may be more challenging to diagnose and treat.

**Objective:**

To describe a cohort of patients with PIDs with atypical presentations of skin infections, who posed a diagnostic and/or therapeutic challenge.

**Methods:**

This is a retrospective study of pediatric patients with PID with atypical presentations of infections, who were treated at the immunodeficiency specialty clinic and the pediatric dermatology clinic at the Sheba Medical Center between September 2012 and August 2022. Epidemiologic data, PID diagnosis, infectious etiology, presentation, course, and treatment were recorded.

**Results:**

Eight children with a diagnosis of PID were included, five of whom were boys. The average age at PID diagnosis was 1.7 (±SD 3.2) years. The average age of cutaneous infection was 6.9 (±SD 5.9) years. Three patients were born to consanguineous parents. The PIDs included the following: common variable immunodeficiency, severe combined immunodeficiency, DOCK8 deficiency, ataxia telangiectasia, CARD11 deficiency, MALT1 deficiency, chronic granulomatous disease, and a combined cellular and humoral immunodeficiency syndrome of unknown etiology. The infections included the following: ulcerative-hemorrhagic varicella-zoster virus (two cases) atypical fungal and bacterial infections, resistant Norwegian scabies, giant perianal verrucae (two cases), and diffuse molluscum contagiosum.

**Conclusions:**

In this case series, we present unusual manifestations of infectious skin diseases in pediatric patients with PID. In some of the cases, recognition of the infectious process prompted life-saving treatment. Increasing familiarity with these dermatological manifestations, as well as keeping a high index of suspicion, is important to enabling early diagnosis of cutaneous infections in PIDs and initiation of prompt suitable treatment.

## 1. Introduction

Primary immunodeficiency disorders (PIDs), also termed inborn errors of immunity (IEI), encompass a variable group of hereditary diseases harboring defects in innate and adaptive immune responses. Patients often present with recurrent infections, failure to thrive, and have an elevated risk of autoimmunity, allergy, and malignancy (1, 2).

Many immunodeficiencies have associated cutaneous eruptions, which can be specific for the disorder or nonspecific. For example, “Eczematous dermatitis,” characterized by erythema, inflammatory papules or plaques, scale, and pruritus, is a common cutaneous presentation of a subset of PIDs [e.g., Omen syndrome, severe combined immunodeficiency (SCID)] (1–5). In addition, primary immunodeficiencies may present with nonspecific inflammatory eruptions, including granulomatous nodules, cold abscesses, urticaria, and ulcers (2, 6, 7).

Infections, common in these disorders, can also manifest on the skin. Some infections have been linked to specific defects such as candidiasis in chronic mucocutaneous candidiasis and autosomal dominant hyper-IgE syndrome (AD-HIES) (8, 9). In addition, severe dermatitis with loss of function of the skin barrier (e.g., AD-HIES) can lead to superinfection with *Staphylococci*, *Streptococci*, *Enterococci*, and *Pseudomonas* manifesting as impetiginization, infectious foci, and cellulitis (10). Due to the inappropriate immune response to unusual but common pathogens, these patients may present with unique infectious manifestations that are not common in healthy individuals and may be more alarming or challenging to diagnose (3, 10). In addition, immunosuppressants given to these patients for various conditions or as an adjuvant to hematopoietic stem cell transplantation can further make them prone to additional infections (11).

This report presents cases of immunodeficient patients with skin infections with unique presentations. Reports of such cases can aid in raising awareness of atypical presentations of skin infections in this population of patients and may help clinicians treating these patients to identify and treat such conditions. The local institutional IRB committee approved the study.

## 2. Materials and methods

This was a retrospective study of all pediatric patients with PIDs with atypical presentations of infections treated at the immunodeficiency specialty clinic and the pediatric dermatology clinic at the Sheba Medical Center, a large tertiary referral center, between September 2012 and August 2022.

Data recorded included the following: epidemiologic variables, personal and family medical histories, laboratory and genetic tests, skin biopsy when feasible, duration of illness, clinical manifestations, treatments prescribed, duration of therapy, clinical response, and side effects.

## 3. Cases

Eight children with a diagnosis of PID were included, five of whom were boys. The average age at molecular diagnosis was 1.7 (±SD 3.2) years. The average age of the atypical infection presentation was 6.9 (±SD 5.9) years.

### 3.1. Diffuse ulcerative-hemorrhagic varicella-zoster virus

#### 3.1.1. Case presentation 1

Case 1 was a 13.5-year-old male, born to non-consanguineous parents of Bukharin origin. He has two healthy siblings. Pregnancy and delivery were uneventful. Skeletal defects and syndromic look [microcephaly, brachycephaly, oligodactyly/ectrodactyly (four toes), camptodactyly, bilateral epicanthal folds, microstomia, and hypodontia] were noted at birth.

From infancy, he was diagnosed with developmental delay, recurrent infections including lung, and joint infection, herpetic infections, and repeated aspirations, eventuating in chronic lung disease.

#### 3.1.2. Immunology

##### 3.1.2.1. Immunoglobulins

IgG-normal (1,020 mg/L), IgA-low (47 mg/L), and IgM-elevated (311 mg/L). Specific serology to IgM and IgG normal. Total lymphocytes-normal, lymphocytic function-functional antibodies were missing as his response to previous vaccination was partially absent despite recurrent immunization. Reduced TREC cells. T cells; CD8 elevated and CD4 decreased (1,723 cells/mm^3^ and 278 cells/mm^3^, respectively). The patient displayed normal T cell function, as determined by response to mitogenic [phytohemagglutinin (PHA) and anti-CD3p stimulations. B cells-normal, NK-normal.

#### 3.1.3. Genetic testing

Chromosomal microarray analysis (CMA), whole exome sequencing (WES), specific gene sequencing, and whole genome sequencing (WGS) did not reveal a pathogenic causative mutation.

#### 3.1.4. Diagnosis

The patient displayed a clinical phenotype (specific infections) suggestive of a syndromic combined (cellular and humoral) immunodeficiency. His blood tests support this diagnosis (a lack of adequate antibody responses, CD4 lymphopenia, and reduced TRECs).

#### 3.1.5. Skin

Manifestations included recurrent episodes of aseptic cutaneous granulomas on his lower limbs, resistant to multiple immunosuppressive treatments progressing to chronic leg ulcers.

Skin biopsy of a nodular lesion on the lower limb at the age of 3 showed extensive lympho-histiocytic aggregates and a few epithelioid cell granulomas. The lymphocytes were CD3/CD8 predominant, indicating an immune dysregulation.

#### 3.1.6. Treatment

The patient was treated with monthly Intravenous Immunoglobulin (IVIG), prednisone (chronic), and mycophenolate mofetil. Past (failed) treatments for the cutaneous granulomas included dapsone, hydroxychloroquine, infliximab, cyclosporine, and methotrexate (MTX).

Due to a chronic course of prednisone treatment, he developed side effects, including growth delay, hypertension, central obesity, cataract, and retinal detachment.

**Figure 1:**
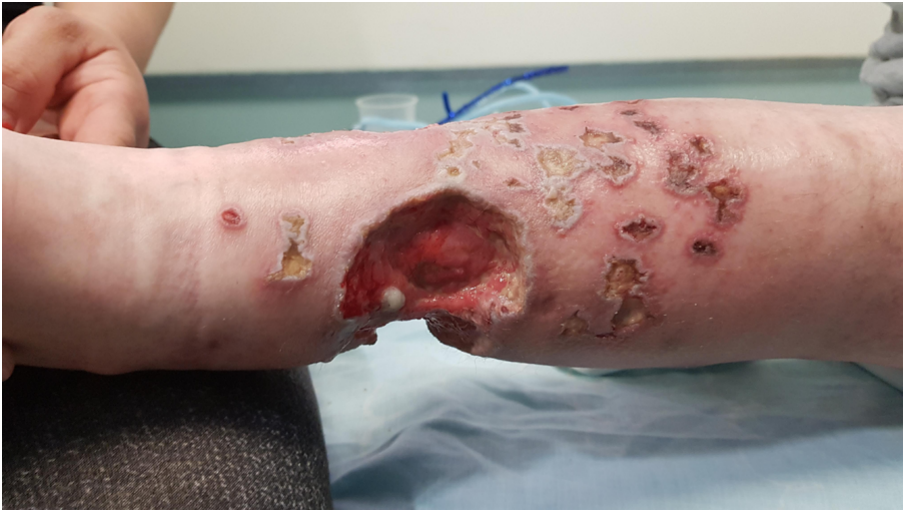
Multiple well demarcated, punched-out, ulcers with erythematous indurated borders, with fibrin on their base and an overlying hemorrhagic crust, on the lower leg of a 13.5-year-old boy with a combined immunodeficiency syndrome, consistent with varicella-zoster virus infection overlying chronic ulcerative granulomas.

At the age of 13.9 years, he experienced a worsening of the lesions on his lower limbs presenting with multiple necrotic hemorrhagic ulcers and vesicles on the lower legs associated with severe pain (Figure 1).

Polymerase chain reaction (PCR) from a skin ulcer and blood was positive for varicella-zoster virus (VZV) and from a hemorrhagic vesicle positive for herpes virus 1 (HSV1). Due to numerous prolonged past courses of both treatment and prophylactic acyclovir, and a concern for possible resistance, foscarnet was started. Most of the small satellite ulcerations healed.

Since the primary ulcer persisted, a tissue culture was taken and *Mycobacterium chelonae* was isolated. The patient was treated with amikacin, clarithromycin, and levofloxacin and showed improvement. Eventually, skin grafting was planned.

Unfortunately, the patient eventually died of candida bacteremia.

#### 3.1.7. Case presentation 2

Case 2 was an 11.5-year-old girl of Arab origin. Her parents are first cousins. She has two healthy sisters.

She was referred to our institute at the age of 9 years for the investigation of a chronic restrictive pulmonary disease, splenomegaly, and lymphadenopathy.

#### 3.1.8. Immunology

Isohemagglutinin-positive anti-A 1:64, anti-B 1:32, TCR double-negative T cells (CD3 + *αβ *+ CD4-CD8-) 3.6%. Direct Coombs-positive. Immunoglobulins, lymphocytes vaccine serology-normal. Neutrophil function, determined by using the Dihydrorhodamine (DHR) test, was normal. IgG levels were normal while on IVIG treatment.

#### 3.1.9. Genetic testing

A genetic test revealed a homozygous *LRBA* mutation, c.3914G > A; p.R1305H (12). The combined annotation-dependent depletion (CADD) score of the mutation suggested pathogenicity.

#### 3.1.10. Diagnosis

The diagnosis was common variable immunodeficiency (CVID), IgA deficiency, and autoimmune thrombocytopenia.

#### 3.1.11. Treatments

The treatment regimen was IVIG (monthly), mycophenolate mofetil, hydroxychloroquine, cyclosporine, trimethoprim–sulfamethoxazole (TMP–SMZ), and bortezomib. Past medications included the following: corticosteroids, rapamycin, rituximab, romiplostim, and abatacept. At the age of 12, the girl eventually underwent successful bone marrow transplantation from a matched related donor.

#### 3.1.12. Skin

**Figure 2:**
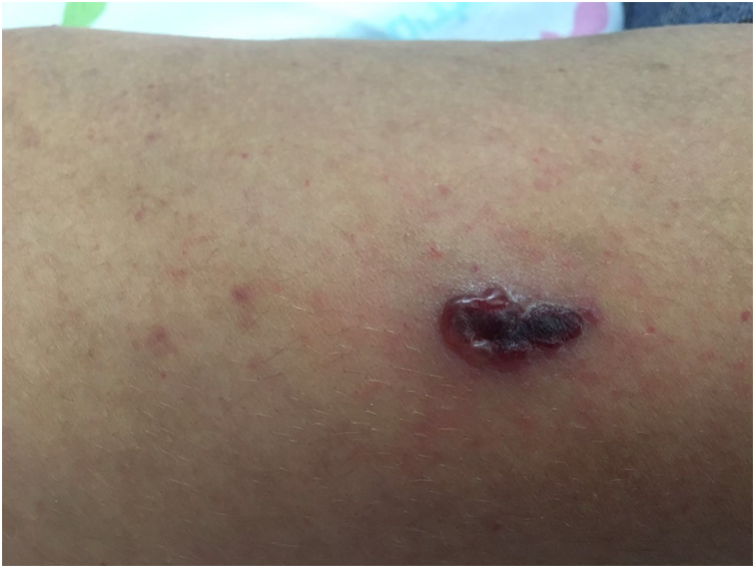
Hemorrhagic vesicle with a necrotic center on the arm of an 11-year-old girl with CVID, consistent with varicella-zoster virus infection. CVID, common variable immunodeficiency.

At age 11, she presented with hemorrhagic vesicles on her trunk and extremities, with a necrotic center (Figure 2). PCR was positive for VZV. Skin biopsy from an ulcer rim on the left thigh showed partially necrotic skin, with a part of the epidermis showing cytological features of herpetic infection. She was treated with IV acyclovir for 8 days and 3 days of oral acyclovir with resolution.

CVID is a heterogeneous disorder characterized by hypogammaglobulinemia and increased susceptibility to recurrent infections, mainly bacterial. Herpetic infections seen in CVID are mainly HSV, VZV, and Cytomegalovirus (CMV). There is an inverse correlation between T lymphocyte count and viral infections (13). In a cohort of CVID patients, CVID patients expressed IgG1 and IgG3 to HSV and CMV like healthy controls, but only IgG1 to VZV. VZV IgG1 and the absence of IgG3 could explain the increased frequency of VZV infections reported in CVID patients (14).

Hemorrhagic varicella is a rare, and at times fatal, complication of chickenpox (12, 15, 16). Other complications of VZV in immunocompromised patients include visceral dissemination, pneumonia, hepatitis, encephalitis, invasive group A streptococcal soft tissue infection, and disseminated intravascular coagulopathy. Early initiation of acyclovir may be life-saving in these patients. Intravenous foscarnet may be required for non-responsive or resistant cases.

### 3.2. Resistant Norwegian scabies infection

#### 3.2.1. Case presentation

A 3.2-year-old male was born to consanguineous parents (first cousins) of Arab origin. A male brother died at age 1.5 years from an undiagnosed condition causing fever and abscesses. A cousin was diagnosed with mucosa-associated lymphoid tissue lymphoma translocation protein 1 (MALT1 deficiency). The patient had three unaffected brothers.

From the age of 5 months, the patient had recurrent episodes of fevers, sinopulmonary infections, and diarrhea. In addition, he suffered from speech delay, poor weight gain, and failure to thrive with colitis diagnosed per colonoscopy.

#### 3.2.2. Immunology

The initial immunologic investigation revealed leukocytosis (26.7 K/μl) with lymphocytosis (16.5 K/μl). Immunophenotyping of lymphocytes showed a normal representation of T, B, and NK cells. The result of the lymphocyte proliferation test was normal following stimulation with PHA and IL-2 but markedly reduced following stimulation with anti-CD3. Peripheral blood mononuclear cells (PBMCs) demonstrated decreased IL-2 expression following stimulation with Phorbol 12-myristate 13-acetate (PMA) and ionomycin. TREC levels were within normal ranges.

#### 3.2.3. Genetics

A novel homozygous MALT1 missense mutation (c.1799T > A; p. I600N) was identified by using WES (17).

#### 3.2.4. Diagnosis

The diagnosis was MALT1 deficiency.

#### 3.2.5. Treatments

The patient was treated with monthly IVIG, prednisone, azithromycin, TMP–SMX, itraconazole, salazopyrin, and inhalations. An identical haploid transplantation from the mother was planned.

#### 3.2.6. Skin

At 11 months, he presented with seborrheic dermatitis and diffuse depigmented macules on the face, trunk, and extremities, diagnosed as vitiligo, which progressed to a widespread distribution including his face, trunk, and extremities. At 12 months, he presented with severe dermatitis; he was treated chronically with topical betamethasone with eventual systemic side effects of the suppression of the hypothalamic-pituitary axis and hirsutism.

At the age of 3 years, he developed an itchy recurrent erythematous papular crusted rash involving the scalp, face, trunk, palms, and soles.

Skin biopsy from a crusted papule on the left lower back showed psoriasiform dermatitis. No parasites were seen, but due to a high clinical suspicion, scabies infection was suspected.

**Figure 3:**
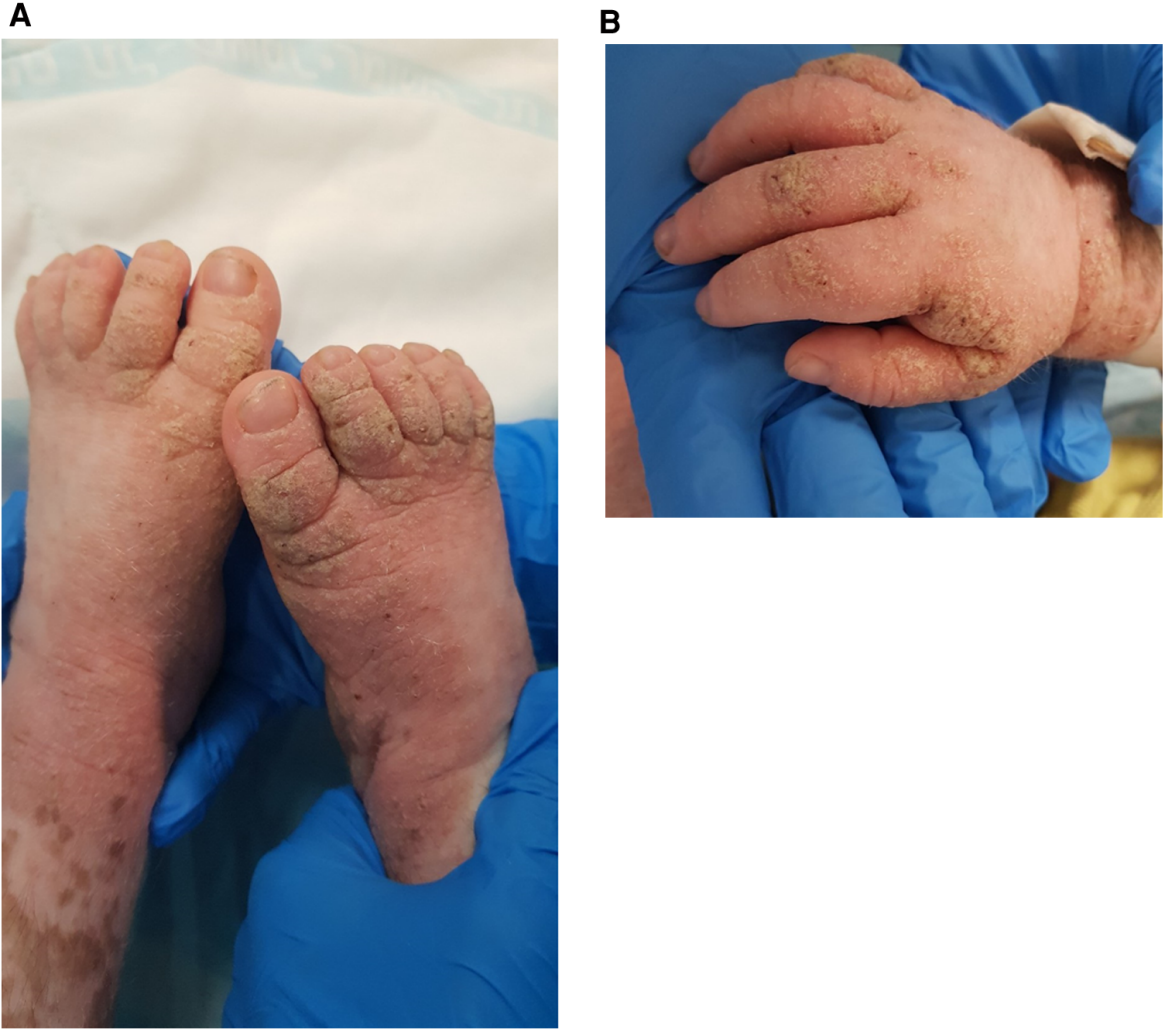
Erythematous plaques with diffuse yellow thick scale and hemorrhagic crusts on the feed (**A**) and hand (**B**) of a 3-year-old boy with MALT1 deficiency, consistent with Norwegian scabies.

The rash was treated with permethrin 5% cream, including the patient's close providers, with improvement but recurrence. A few courses of permethrin were given, with only partial response. Then, he was treated with sulfur 10% ointment and a single dose of oral ivermectin of 200 μg/kg. Rash recurred and progressed to thick hyperkeratotic plaques on the extremities, palms, and soles (Figure 3A,B). Scraping from the keratotic skin lesions was positive for *Sarcoptes scabies*, and Norwegian scabies was diagnosed. Of note, his caretakers had a complete response to the treatment but were reinfected. Two courses of ivermectin 400 μg/kg were given 10 days apart with intense keratolytic treatment of sulfur 10% and salicylic acid without marked improvement.

MALT1-deficient patients showed defective signaling characterized by impaired NF*κ*B activation and IL-2 secretion, and MALT1-deficient patients exhibited reduced regulatory T cells (Tregs) and T helper 17 (TH17) cells. Only several cases of MALT1 deficiency have been reported to date (18, 19).

Patients with MALT1 deficiency have been characterized by a combination of recurrent bacterial, viral, and fungal infections. Notably, sepsis and meningitis were the most common severe infections (20). Severe scabies infection was not previously reported. MALT1 is required for the generation of TH17 cells, and low levels of Th17 cells were found in MALT1-deficient patients (21). However, crusted scabies is associated with TH2-skewed immune reaction and increased and not decreased production of Th17 cytokines (22). Therefore, the etiopathology of resistant crusted scabies in these patients remains to be studied.

### 3.3. Atypical fungal infection

#### 3.3.1. Case presentation

An 11-year-old girl of Arab origin was diagnosed with ataxia telangiectasia (AT) at 1 year of age. Her parents are first cousins; both are healthy. She has four siblings, one of which was diagnosed with AT, as well as some other family members.

In her early childhood, the patient suffered from ataxia and repeated lung and ear infections. She also developed sclerosing cholangitis and hypothyroidism. In later years, she used a wheelchair for mobility. Eventually, she was diagnosed with diffuse large B cell lymphoma.

#### 3.3.2. Immunology

Lymphocyte count and function-normal, decreased immunoglobulins-IgG (516 mg/L).

#### 3.3.3. Treatments

Monthly IVIG, inhalations (budesonide, fluticasone propionate), and levothyroxine.

#### 3.3.4. Skin

Multiple café-au-lait spots and telangiectasias.

At 11 years, the patient developed a spreading skin rash on her nose. The lesions spread to the nostrils and her left cheek.

Initially, she was treated with amoxicillin and clavulanic acid and a topical antibiotic (chloramphenicol), however, with no response.

**Figure 4:**
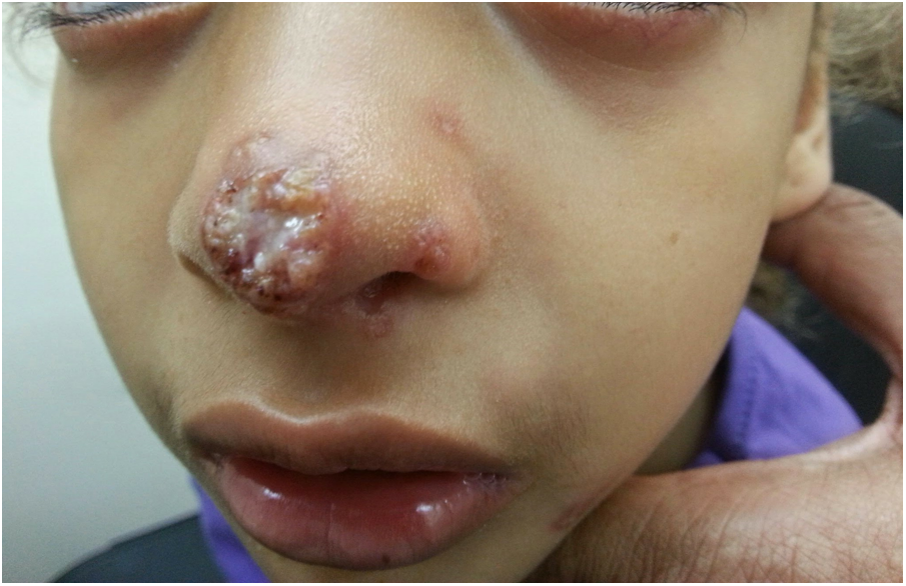
An erythematous plaque with a central erosion, circinate-elevated border, overlying pustules, and purulent discharge on the tip of the nose of a 10.5-year-old girl with AT. Small satellite papules were also present on the nostrils, consistent with a fungal infection. AT, ataxia telangiectasia.

Upon examination, on the tip of the nose, there was an erythematous plaque with a central erosion and a circinate-elevated border with overlying scattered pustules. The nose bridge and nostrils had small erythematous erosions (Figure 4).

An atypical infection was suspected. The result of a PCR test for *Leishmania* was negative. A mycological test showed hyphae, but a species could not be identified due to a small sample.

She was then treated with oral fluconazole 5 mg/kg for 4 weeks and a topical combination antibiotic and antifungal treatment (ciclopirox, gentamicin) with a good response but with remaining lesions in the nostrils. At this point, erythematous plaques with a gold crust were seen around the nostrils and impetigo was suspected. Repeated cultures grew *Staphylococcus aureus* and *Candida*.

Fluconazole, cephalexin, and nasal bacitracin were given, with improvement seen in the patient’s condition, but frequent impetigo recurrences followed. She was therefore started with prophylactic treatment of cephalexin for 1 month with resolution and no recurrences.

AT patients rarely acquire opportunistic infections, although they may be more susceptible to viral infections (HSV, VZV, Human papillomavirus (HPV) warts, molluscum). Their T cell function may be relatively intact, thus explaining the uncommon occurrence of opportunistic infections (23). A case report described an AT patient with an invasive candida infection due to a functionally impaired T cell function (24). Our case was a diagnostic challenge. Due to residence in an endemic region, leishmaniasis was initially high on the differential, but eventually, clinical suspicion and fungal cultures were of help.

### 3.4. Atypical bacterial and candida infection

#### 3.4.1. Case presentation

Another case was a 2-year-old girl whose parents are healthy and are first cousins of Arab origin. She has three healthy siblings. Two more brothers died from infections at the age of 6, one of whom had allergies, angioedema, and eczema.

From the age of 1 month, the patient had recurrent ear and herpetic infections. In addition, she had hepatosplenomegaly and failure to thrive. But she had no known allergies.

#### 3.4.2. Immunology

Immunoglobulins-normal, IgE-elevated (4,310–135 K mg/dl), lymphocytes immunophenotyping;CD4+ CD3−. TCR-mild restriction polyclonal, decreased TREC (190 copies).

#### 3.4.3. Genetics

A homozygous mutation in *DOCK8* was identified by using WES and designated c.5134C > A; p.S1711X (25).

#### 3.4.4. Diagnosis

DOCK8 immunodeficiency syndrome.

#### 3.4.5. Skin

At the age of 5 months, the girl presented with a widespread eczematous rash. A biopsy showed spongiotic psoriasiform dermatitis.

#### 3.4.6. Treatment

She was treated with prednisone, topical corticosteroids, and antihistamines. TMP–SMZ, fluconazole, and acyclovir were also given. At 1 year and 9 months of age, the patient received Hematopoietic Stem Cell Transplantation (HSCT) from her brother.

**Figure 5:**
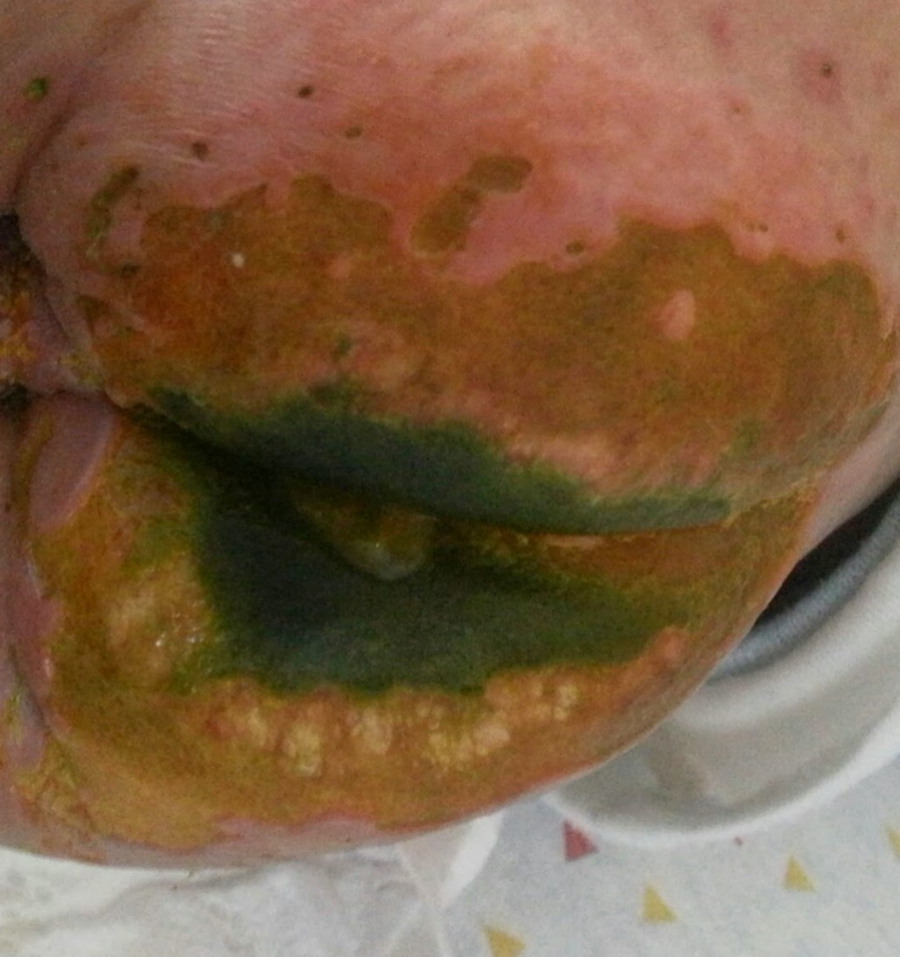
Widespread ulceration on the buttocks and scrotum with a central black eschar in their center and yellow-green exudate in a 5-month-old girl with DOCK8 deficiency, consistent with *Candida* and *Pseudomonas* infection.

One month after the HSCT, she presented with ulcerations on the buttocks, inguinal areas, and lower limbs. Ulcers on the thigh had black eschars in their center, and on the buttocks, the ulcers had green exudate (Figure 5). Tissue culture showed a mixed bacterial growth of three colonies (gram-negative bacilli and gram-positive cocci) and yeast. Blood cultures grew *Pseudomonas aeruginosa* and *Candida krusei*. She was treated with piperacillin/tazobactam, amikacin, and amphotericin B. Topical treatment included silver sulfadiazine and advanced dressings. Unfortunately, the patient died of septicemia.

AD-HIES syndrome is a rare immunodeficiency caused by STAT3 mutation and characterized by eczema, *Staphylococcus aureus* skin abscesses, pneumonia with pneumatocele formation, *Candida* infections, and skeletal/connective tissue abnormalities (26).

DOCK8 deficiency and AD-HIES are characterized by recurrent bacterial and viral infections, atopic eczema, and increased serum IgE levels (27).

The cutaneous viral infections, the most striking and distinguishing feature of DOCK8 deficiency, are extensive, difficult to control, and often occur concurrently. The most common viruses involved are HSV, HPV, molluscum contagiosum (MCV), and VZV (28).

We encountered a combined candida and pseudomonas severe infection in a patient with DOCK8 deficiency, which eventually resulted in death.

In this case, the pseudomonas and candida infections were likely related to the increased immunosuppression from the HSCT, the antirejection medications, and neutrophil dysfunction, given the close proximity of the infection to the transplant.

### 3.5. Giant diffuse verrucae

#### 3.5.1. Case presentation 1

This patient was a 16.5-year-old boy born to healthy non-consanguineous parents. Two maternal uncles died in infancy. He has two healthy siblings. From infancy, he had recurrent infections, including skin abscesses, herpetic infection, and an episode of bacteremia.

#### 3.5.2. Immunology

Tests prior to HSCT at 0.5 years of age were not available (performed at another hospital).

#### 3.5.3. Genetic testing

A genetic test showed a common gamma chain (IL2Rγ) hemizygote mutation designated c.786_787 delins T; p.V263fsX10, and the mother is a carrier of the mutation.

#### 3.5.4. Diagnosis

X-linked SCID.

#### 3.5.5. Treatment

IVIG was given only on a monthly basis, and haploidentical stem cell transplantation was done at 0.5 years of age. Following the transplant, there was only partial immune recovery, and NK cells were not recovered.

#### 3.5.6. Skin

**Figure 6:**
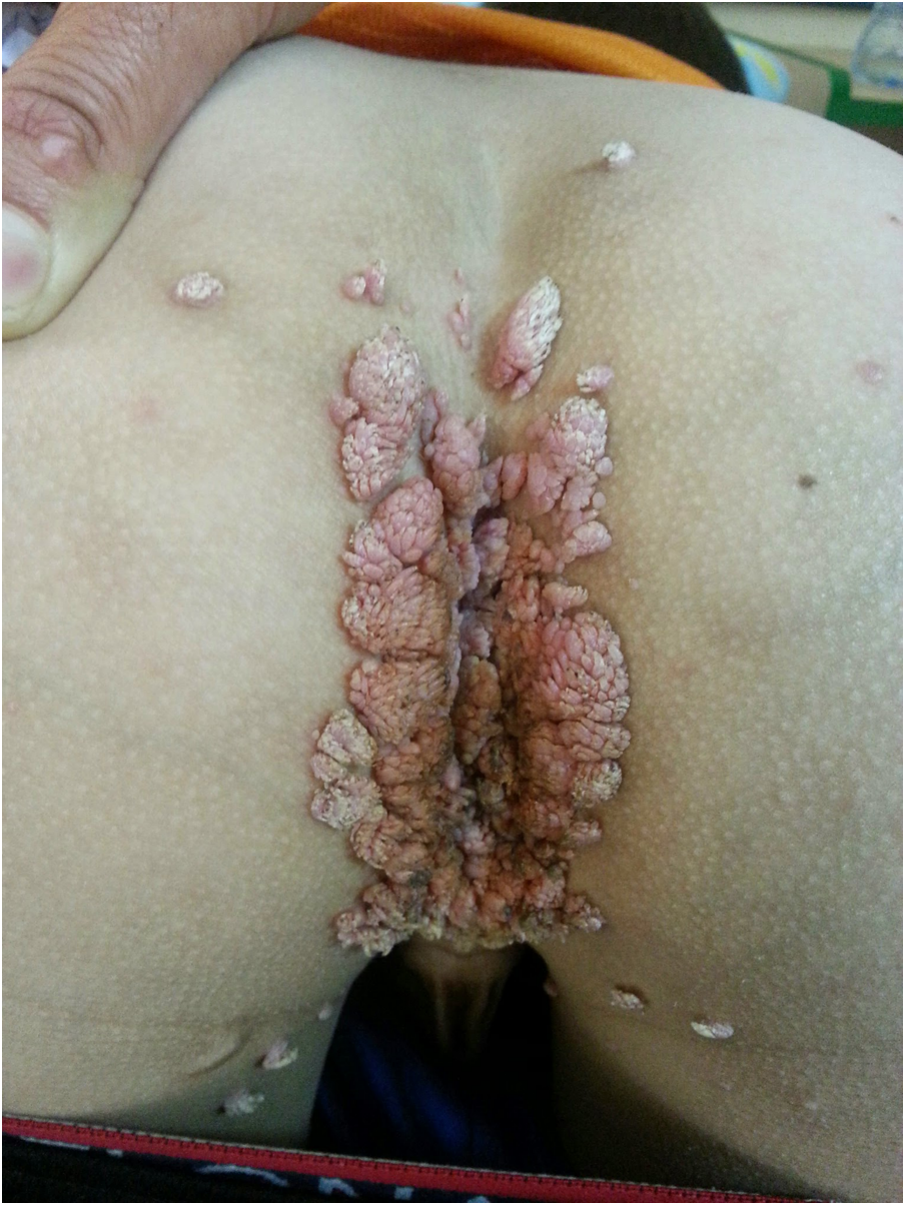
Diffuse widespread pink-gray verrucous exophytic papules and plaques on the perianal and perigenital regions of a 14-year-old boy with X-linked SCID, consistent with condyloma acuminata. SCID, severe combined immunodeficiency.

From the age of 14, the patient suffered from diffuse verrucae. First, they appeared on only one finger but then spread to the other fingers and the perianal area, genitals, and face (Figure 6). The result of HPV staining from one of the lesions was positive.

The patient was treated with liquid nitrogen every 2 weeks, imiquimod for a few months, and Pulsed dye laser (PDL) laser with partial response and recurrence. The laser procedure proved traumatic, with a painful recovery. The perianal lesion caused pain with defecation. Topical cidofovir, 3% twice daily, was applied, resulting in partial response and local irritation.

The patient’s monthly IVIG dose had a therapeutic effect on his verrucae. He continues to have verrucae but to a lesser degree.

SCID is a rare primary deficiency of T cell infections that can be severe. Allogeneic hemopoietic stem cell transplantation, or gene therapy in some cases, is a life-saving treatment for these patients. Yet, some patients who undergo HSCT develop late infections because of insufficient or declining immune function (29).

In a study of 177 infants with SCID who underwent HSCT, half were given IVIG. Post-transplant, 40% of the patients had HPV warts; 81% had genetic defects associated with poor or reduced NK-cell function. A total of 41% reported warts ranging in severity from a transient infection to a persistent medical problem, affecting their quality of life (30). Our patient with SCID showed a late onset (13 years) of HPV warts after HSCT, as described in a cohort of 44 patients who had HPV warts at a median age of onset of 8 years after transplantation (29).

#### 3.5.7. Case presentation 2

This patient was a 5-year-old boy born to healthy, non-consanguineous parents of Ukrainian and Romanian origin. He has one healthy sister.

Since infancy, he was diagnosed with atopic dermatitis and sesame allergy.

At 1 year of age, he had repeated episodes of fevers, neutropenia, aphthous stomatitis, and elevated inflammatory markers; also recurrent viral illnesses.

#### 3.5.8. Immunology

Lymphocyte count and function-normal, Immunoglobulins-normal Immunophenotyping of lymphocytes phenotyping-normal T, B, and NK cells; vaccine response-normal; autoantibodies for neutrophils-weak positive. A panel of autoantibodies was found negative. Neutrophilic function was dysfunctional.

#### 3.5.9. Genetic testing

A heterozygote mutation R47H in the caspase activation and recruitment domain family member 11 (CARD11) were identified in a genetic test.

#### 3.5.10. Diagnosis

CARD11 deficiency.

#### 3.5.11. Skin

**Figure 7:**
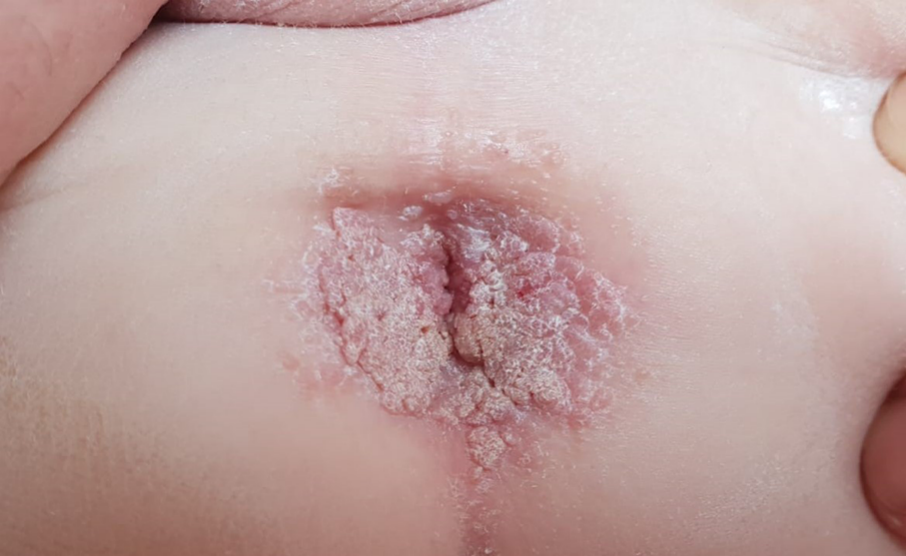
Diffuse pink-gray verrucose papules on the perianal area of a 1.5-year-old boy with CARD11 deficiency, consistent with condyloma acuminata. CARD11, caspase activation and recruitment domain family member 11.

At 1 year and 9 months of age, the patient had verrucose exophytic papules and plaques on the perianal area, with gradual growth and outward expansion to the surrounding skin (Figure 7). The HPV staining result was positive. Both parents had no clinical signs or past history of condylomas. Condyloma acuminata was diagnosed. He was treated with imiquimod three times a week for 6 weeks, showing good response and a resolution of the lesions. He had viral symptoms (mild headache and muscle weakness) a day after the application and moderate local irritation, which was tolerated with topical steroid treatment.

CARD11 encodes a structural protein in lymphocytes that links antigen receptor engagement with downstream signaling to nuclear factor *κ*B (NF*κ*B). Patients with CARD11 deficiency present with atopy, viral skin infections, and/or respiratory infections and exhibit defective TCR-induced NF*κ*B activation *in vitro* (31).

Cutaneous viral infections (e.g., molluscum, HSV-1) are common in CARD 11-deficiency patients. Impaired CD8+ T cell immunosurveillance could be a causative factor and may also help explain tumor development in certain patients (32). Diffuse cutaneous warts are common in epidermodysplasia verruciformis (EV) GATA deficiency and warts, hypogammaglobulinemia, infections, and myelokathexis (WHIM) syndrome and may sometimes be suspected initially (33), but other PIDs can also manifest with diffuse HPV warts such as SCID (described above), DOCK 8 deficiency, and Netherton syndrome (3).

Perianal warts in young children with immune deficiency represent a therapeutic dilemma. Treatment options include cryotherapy, curettage laser, podophyllin, and tri/bichloroacetic acids. However, these treatments are painful and immunosuppressive patients are prone to infectious complications and abnormal healing. Our patient underwent a successful treatment with imiquimod, a topical immunomodulator. There are a few reports on the use of imiquimod in this age group (34–36), however, none on its use in immunodeficient patients.

### 3.6. Diffuse molluscum contagiosum

#### 3.6.1. Case presentation

This patient was a 1.5-year-old boy of Arab ethnicity, born to healthy non-consanguineous parents, and has one healthy older sister.

From early infancy, the patient experienced failure to thrive, had milk allergy with bloody stools, lymphadenopathy, and splenomegaly.

#### 3.6.2. Immunology

Elevated immunoglobulins, T and B cell numbers were normal (4,821 and 3,304 cells/mm^3^, respectively); test result of Bacillus Calmette–Guéri (BCG) vaccine-positive (The patient had axillary lymphadenopathy, which was positive to BCG.). There was altered neutrophil function as determined by using the DHR test.

#### 3.6.3. Genetic testing

A hemizygote CYBB (GP91) stop codon mutation (c.388C > T, p. Arg130Ter) was found.

#### 3.6.4. Diagnosis

Chronic granulomatous disease (CGD) X-linked, BCGitis.

#### 3.6.5. Treatment

TMP–SMZ, itraconazole, tuberculosis (BCGitis)-triple antibiotic therapy with isoniazid, rifampin, and ethambutol.

#### 3.6.6. Skin

**Figure 8:**
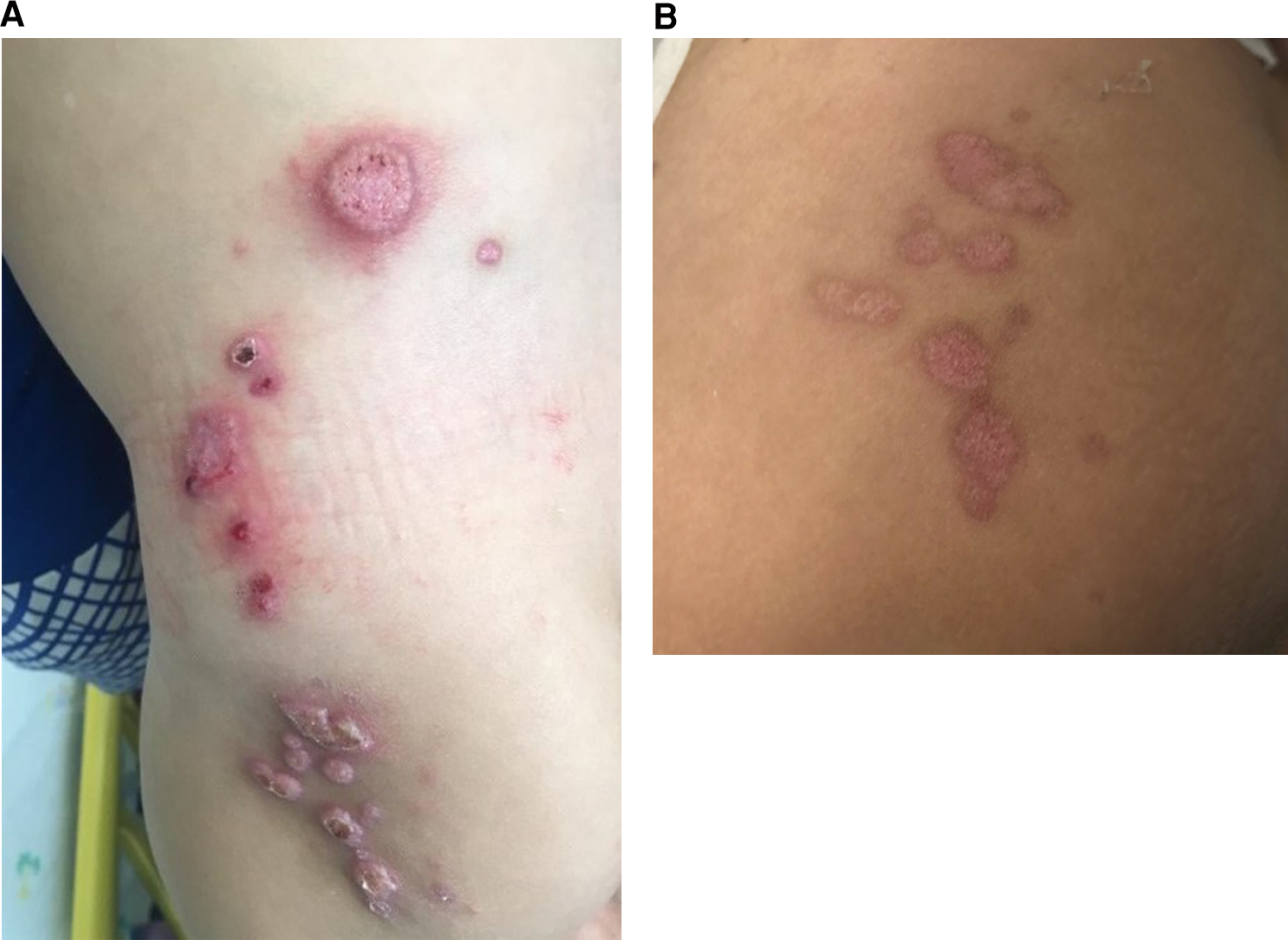
(**A**) Erythematous papulonodular eruption on the buttocks, lower back, and hip with scale and hemorrhagic crusts in a 10-month-old boy with X-linked CGD, consistent with molluscum contagiosum. (B) Post-treatment with IV cidofovir and a resolution of the lesions with the remaining postinflammatory changes and mild scarring. CGD, chronic granulomatous disease.

At 10 months of age, the patient presented with erythematous papules and nodules on the buttocks, which spread to the inguinal areas and his back (Figure 8A). In addition, he had an erythematous purulent nodule in the left inguinal region and bilateral lymphadenopathy. The clinical differential diagnosis included inflammatory conditions (granulomas, sarcoidosis) or an atypical infection (mycobacterial, fungal, or viral).

Skin biopsy—(1) suppurative dermatitis with scale crust and epidermal micro-abscess formation. Periodic acid- Schiff (PAS), Giemsa (GMZ), Ziehl- Neelsen (ZN), and HSV stains were negative. (2) Consistent with MCV. PCR test result was positive for MCV.

Culture of pus drained from a left inguinal abscess grew *Klebsiella pneumonia*.

An ultrasound of the inguinal area was notable for left inguinal phlegmon with hyperemic lymph nodes up to 20 mm.

The patient was treated with weekly cidofovir (5 mg/kg) for 4 weeks, IV vancomycin and piperacillin–tazobactam, and topical antibiotics with good response (Figure 8B).

CGD is characterized by the absence or malfunction of the Nicotine adenine dinucleotide phosphate (NADPH) oxidase in phagocytic cells. CGD patients suffer from recurrent, life-threatening bacterial and fungal infections. There are limited reports of severe viral diseases such as those reported by us in this series. Bone marrow stem cell transplantation is currently the only curative treatment (37).

## 4. Discussion

In this case series, we present unusual manifestations of common infectious skin diseases in pediatric patients suffering from diverse types of PIDs.

Skin infections are among the most prevalent dermatologic manifestation of PIDs (38). An extensive systematic review analyzed skin disorders and their prevalence in PIDs (39). Sixty-seven articles (5,030 patients) were included. Skin infections, including bacterial, fungal, and viral infections, showed high prevalence in combined immunodeficiencies with associated or syndromic features such as autosomal recessive hyper-IgE syndromes and diseases of immune dysregulation, whereas autoinflammatory disorders and complement deficiency showed lower prevalence. Among skin infections, viral infections with VZV and HSV had the highest prevalence in PID (3, 39).

The patients in our case series required evaluation by a multidisciplinary team comprising pediatric dermatologists, pediatric infectious disease specialists, immunologists, and others so that a correct diagnosis and treatment could be achieved. Inpatient consultative pediatric dermatology represents a unique subspecialty, but data on dermatological hospital consultations and the management of pediatric patients are limited (40, 41). In our tertiary medical center, The Edmond and Lily Safra Children's Hospital at the Sheba Medical Center, the Pediatric Dermatology Service includes three pediatric dermatologists who provide consultation to 13 inpatient departments with a total of 20,000 admissions per year. As a referral center for PID patients following up ∼700 patients with a variety of PIDs, we have obtained wide experience with infectious as well as non–infectious cutaneous manifestations of this patient group (42).

We hereby report eight cases of various immunodeficient patients with a unique presentation of skin infections, which posed a diagnostic and therapeutic challenge. In some patients, recognition of the infectious process prompted the administration of life-saving treatment. Therefore, ensuring increased levels of familiarity with these dermatological manifestations, as well as keeping a high index of suspicion, is important to facilitate an early diagnosis of cutaneous infections in PIDs.

We highlight that immediate and easy accessibility to dermatological consultation, including advanced evaluation (skin biopsy and immunohistochemistry, skin cultures, etc.), provides an advantage for the management of such patients with complex conditions. Furthermore, this series and other reports may help clinicians treating patients with PIDs to identify and treat such infections promptly.

## Data Availability

The raw data supporting the conclusions of this article will be made available by the authors, without undue reservation.
